# Iron Deficiency in Young Children: A Risk Marker for Early Childhood Caries

**DOI:** 10.5005/jp-journals-10005-1176

**Published:** 2013-04-26

**Authors:** Pushpa Iranna Koppal, Mohan Ravishankar Sakri, Basavaprabhu Akkareddy, Dharam M Hinduja, Raviraj Annayya Gangolli, Basanagouda C Patil

**Affiliations:** Senior Lecture Department Pedodontics and Preventive Dentistry S Nijalingappa Institute of Dental Sciences and Research Centre Gulbarga, Karnataka, India; Reader, Department of Conservative and Endodontics, Mansarovar Dental College, Bhopal, Madhya Pradesh, India; Reader, Department of Pedodontics and Preventive Dentistry, Sinhgad Dental College and Hospital, Pune, Maharashtra, India; Reader, Department of Conservative and Endodontics, Government Dental College and Hospital, Bengaluru, Karnataka, India; Reader, Department of Pedodontics, KGF College of Dental Sciences and Hospital, Kolar, Karnataka, India; Reader, Department of Orthodontics, S Nijalingappa Institute of Dental Sciences and Research Centre, Gulbarga, Karnataka, India

**Keywords:** Iron deficiency, Early childhood caries

## Abstract

**Aim:**

1. Evaluate the coexistence of iron deficiency and early childhood caries.
2. Evaluate whether iron deficiency can be considered as a risk marker for early childhood caries.
3. Estimate the incidence of iron deficiency in children with early childhood caries.
4. To evaluate and compare the iron status of children with and without severe early childhood caries.

**Materials and methods:** Sixty children of age 2 to 6 years in whom blood investigations are advised by pediatricians are selected for the study and are divided into early childhood caries (ECC) and control groups according to the def index. After obtaining the informed consent from parent, blood investigations are carried out in these children for the estimation of iron status.

**Results:** All the values depicting the iron status are found to be decreased in the clinical trial group (ECC group) and they are statistically significant.

**Conclusion:** Iron deficiency is observed definitely in children having ECC.

**How to cite this article:** Koppal PI, Sakri MR, Akkareddy B, Hinduja DM, Gangolli RA, Patil BC. Iron Deficiency in Young Children: A Risk Marker for Early Childhood Caries. Int J Clin Pediatr Dent 2013;6(1):1-6.

## INTRODUCTION

Childhood is an important stage in child's life and early childhood caries are the most common disease of this phase. This is often accompanied by serious comorbidities affecting children, their families, the community and the health care system.^1^

Iron deficiency being the most common nutritional deficiency in childhood is often seen associated with severe caries destruction.^2^ Lack of iron is one of the most common dietary deficiency observed worldwide particularly in developing countries. In some instances this deficiency is alleviated by supplementary foods with added iron salts. In many countries where the iron deficiency is apparent, the prevalence of dental caries is high.^3^

There is a substantial body of research that shows a strong association between the frequency of sucrose ingestion and the development of caries in children.^3^ High sucrose diet is of concern because it has been suggested that such diets may be low in micronutrients and compromise nutrient intake. The physicians and dentist, treating young children, should consider that caries in young children is a risk marker for undernutrition.^4^

## MATERIALS AND METHODS

### Subjects

**Figs 1A to D:**
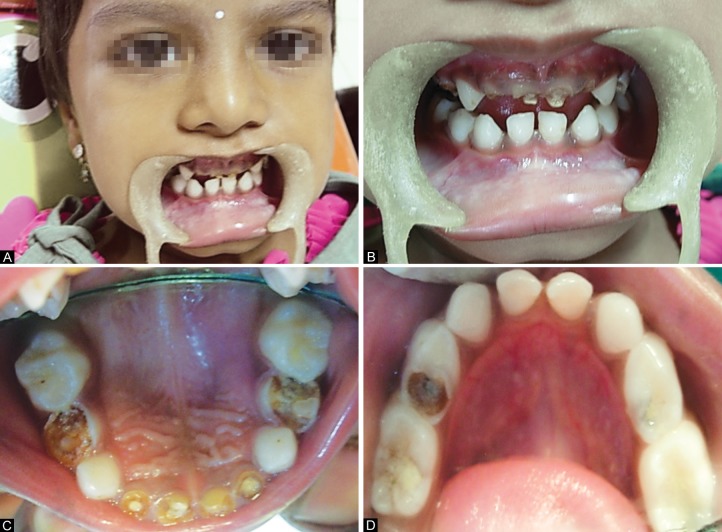
Intraoral photographs of a child with S-ECC

Sixty children of age 2 to 6 years old for whom blood nvestigations were advised, were selected from Outpatient Departments of PMNM Dental College and Hospital, Bagalkot, Outpatient Departments and Inpatient Departments of SN Medical College and Hospital, Bagalkot and Private Pediatric Hospitals of Bagalkot. These children were screened for the presence or absence of severe early childhood caries according to the American Academy of Pediatric Dentistry Guidelines^5^ (Figs 1 and Figs 3).

### Data Collection

Ethical clearance was obtained from the relevant human research ethics committees. The procedures and possible benefits were explained to the parents or guardians of the human subjects involved and their informed consent was obtained prior to the investigation.

**Figs 2A and B:**
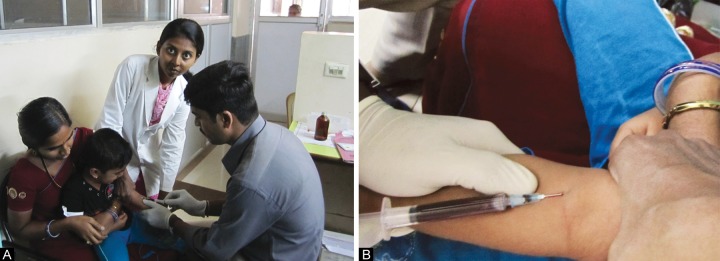
Phlebotomy (collection of blood sample from a child with S-ECC)

After the dental examination was completed, when the children underwent blood investigation, blood was withdrawn from them by phlebotomy,^6^ maintaining all the aseptic precautions (Figs 2 and Figs 4).

Collected blood sample was added to k2/k3 EDTA vacutainer for estimation of hemoglobin and mean corpuscular volume (MCV) and was also transferred to the sterile test tubes with barcodes which was transported to Thyrocare laboratory for the estimation of serum ferritin. Thyrocare is the clinical chemistry laboratory in India. It is the first ISO 9001-2000 certified clinical chemistry laboratory. From 2004, it has been certified by NABL (National Accrediation Board for Testing and Calibration Laboratories), the premium technical body for accreditation in the country under the aegis of Department of Science and Technology, Government of India. Adding one more feather to the cap, since 2007, it has been accredited by the College of American pathologists (CAP), a global accreditation organization.^7^

Thus, blood sample was used to determine the most remarkable blood tests to detect iron deficiency.^4^ They are

- Serum ferritin
- Hemoglobin estimation
- Mean corpuscular volume

Control and clinical trial (early childhood caries group) groups were compared for iron status of the body.

### Analysis of Data

All these children were divided into the following groups:

- According presence or absence of caries as
  - Group without severe early childhood caries (ECC) as control
  - Group with severe ECC as clinical trial group
- According to serum ferritin and hemoglobin (Hb values children were categorized as iron deplete and normal^8^
  Iron deplete: Serum ferritin ≤10 μg/1
  Normal: Serum ferritin ≥10 μg/1

**Figs 3A to D:**
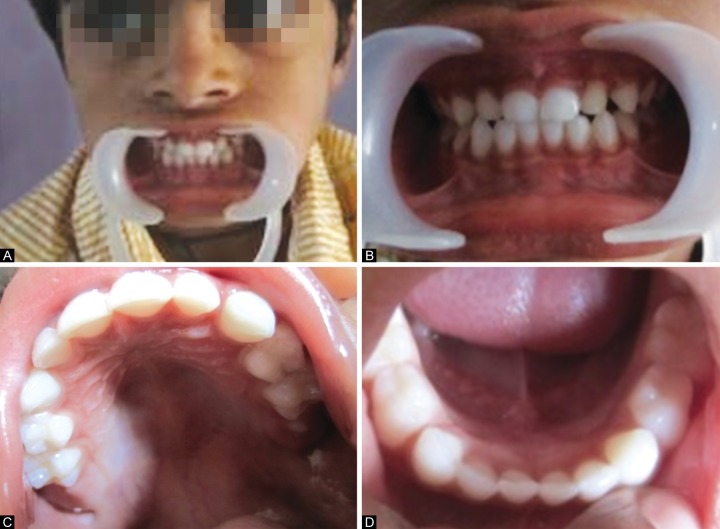
Intraoral photographs of a child without S-ECC

**Figs 4A and B:**
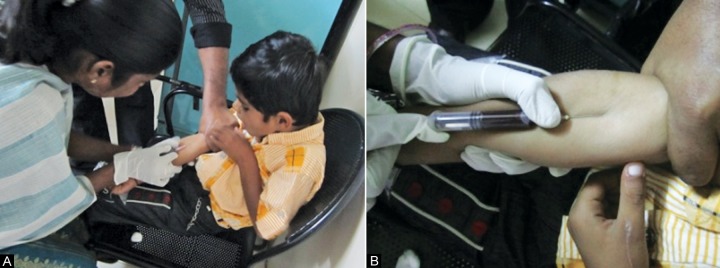
Phlebotomy (collection of blood sample from a child without S-ECC)

## RESULT

Among the 60 children 30 were in control and 30 were in clinical trial group. It was found that children with iron deplete status were more in clinical trial group, i.e. early childhood caries group than control group.

## DISCUSSION

Iron deficiency is most common among the malnourished children and most commonly seen in the age group of 2 to 3 years. Children are most vulnerable to this condition due to their socioeconomic status, lack of education, poor sanitization and increased demand during rapid growth in childhood.^9^

In our study, we compared the iron status of a normal child with that of the child with severe early childhood caries.

**Graph 1:**
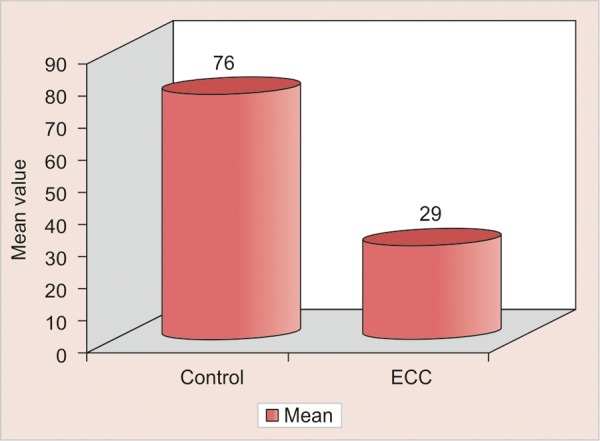
Comparison of control and ECC groups with respect to serum ferritin values

The level of serum ferritin of control group is significantly higher than ECC group (Table 1 and Graph 1). This was in accordance with the hypothesis that prolonged bottle-feeding, which is a risk factor for ECC, is associated with a lower intake of iron, perhaps mediated by bottle-fed children's overconsumption of iron poor foods, such as milk, displacing their desire for foods with a greater iron content.^10^

Low ferritin is evidence that the body has depleted storage of iron in an effort to maintain hemoglobin at an appropriate level for good health. The results suggest that this mechanism was activated to some degree in the S-ECC population because these were children with acceptable levels of hemoglobin but low levels of serum ferritin.^10^

**Graph 2:**
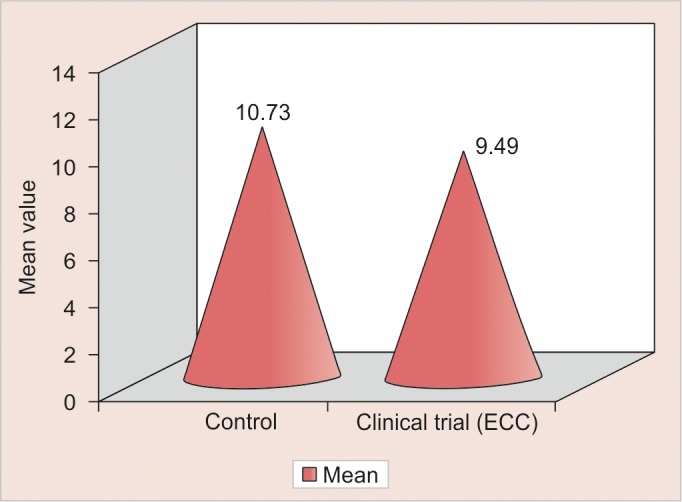
Comparison of control and clinical trial (ECC) groups with respect to Hb (gm/dl) values

Value for hemoglobin of control group is significantly higher than ECC group (Graph 2). This could be related to the population-based study done by Looker et al and most notable, that there was a significant proportion of children with unacceptably low levels of both hemoglobin and ferritin. This finding suggests that the children had insufficient dietary intake of iron to maintain acceptable levels of either hemoglobin or serum ferritin.^10^

**Table 1:** **Table 1:** Comparison of control and ECC groups with respect to serum ferritin values by t-test

*Group*	*Mean*	*SD*	*t-value*	*p-value*
Control	76.0547	84.7486	2.9023	0.0052*
Clinical trial(ECC)	29.3390	24.2945		

*p < 0.05

**Graph 3:**
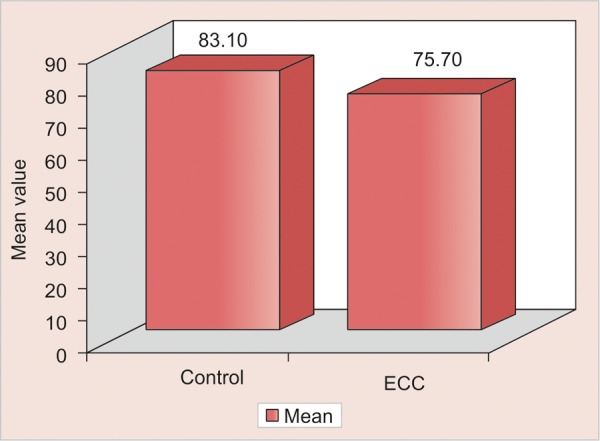
Comparison of control and ECC groups with respect to mean corpuscular volume

Data regarding MCV of control group was also significantly higher than ECC group (Graph 3). MCV is the red blood index used to detect the iron deficiency, anemia. Prolonged bottle-feeding is a marker of clinical significance because of its association with iron deficiency.^10^

Among the 60 children 30% of children in ECC group are iron deplete. Whereas 10% of children in control group are iron deplete (Table 2). This could be due to the common explanatory variables for early childhood caries and iron deficiency, i.e. prolonged bottle-feeding has significant associations found with tooth decay and iron deficiency anemia.^8^

Prolonged bottle-feeding, past 2 years of age; are associated with iron deficiency in South-east Asian toddlers.^11^

**Table 2:** **Table 2:** Distribution of iron deplete status in ECC and control groups

*Group*	*Iron deplete*	*%*
Control	3	10.00
Clinical trial (ECC)	9	30.00
Total	12	20.00

This can be explained because there is significant association between prolonged bottle-feeding and bottle-feeding at sleep time and the incidence of ECC.^12^ Thus, the common relation between the ECC and iron deficiency is prolonged bottle-feeding.^13^

Acs et al reported a relationship between ECC and failure to thrive – a condition of poor growth in young children in a cohort of low income children. This study suggests that the manifestations of nursing caries may go beyond pain and infection. Although, pain and infection may be the primary effects of nursing caries the condition also may effect general health. They concluded that children with nursing caries weighed significantly less than control children.^14^

Graham et al gave the conclusions and implications that the cultural influences that prolong bottle feeding past 2 years of age are associated with iron deficiency in Southeast Asian toddlers. Weaning with a cup, with increased solid food intake should be encouraged by 18 months of age. When this is not accomplished, toddlers should be monitored for development of iron deficiency and oral iron supplements should be provided.^11^

Bowen studied biological mechanisms of ECC. In his study, he has mentioned the effect of dietary metals on ECC. Many children who have had extensive caries were malnourished. Some of these children have iron deficiency anemia, which results in reduced salivary flow in experimental animals. This effect is independent of any topical effect that iron has prevention on caries.^2^

Clarke et al suggest that severe ECC may be a risk marker for iron deficiency anemia. Since, iron deficiency has permanent effects on growth and development, pediatric dentists should recommend assessment of iron levels in severe ECC patients regardless of their anthropometric appearance.^4^

Iron has a cariostatic effect. Iron is able to cover enamel surface with the protective layers. Acid protective layer effect has been demonstrated *in vitro*. It can be assumed that these protective layers will be firmly bound to the organic parts of the enamel as hydrous iron oxides and clay materials have great affinity to organic materials. This affinity has been utilized in the clinical work to increase the adhesion between the composite resins and dentin by mordanting with ferric chloride. Gels and the crystals of hydrous iron oxides are capable of adsorbing various ionsand of nucleating various crystalline substances. From a dental point of view, the absorption of calcium and phosphate ions indicates that iron may have repairing function. This can be by nucleating the precipitation of salivary calcium and phosphate ions as apatite or other phosphates on the enamel surface. Therefore, the iron may have the effect of replacing minerals which can have been dissolved during the acid phases of the caries process. In addition, owing to the above-mentioned affinity to organic materials iron ought to be able to mediate the fixation of remineralized particles to the organic parts of the enamel.^15^

Though we have found the values of variables depicting iron status are low for ECC group compared to control group, these values cannot be used as a risk marker to predict ECC, because ECC is a multifactorial disease. ECC and iron deficiency are interrelated but to predict as a risk marker may require a longitudinal study beginning at the age of eruption of first primary tooth and requires follow-up. The other limitations are analysis was limited because the comparison with reference values required the categorization of the data into age, gender and percentile groups. This resulted in subcategories that were too small for accurate statistical testing of groups or means. Thus, we concluded that both the diseases are multifactorial and the direct relation between the ECC and iron deficiency could not be drawn but iron deficiency and ECC are definitely interrelated.
